# Natural Selection and Adaptive Evolution of Leptin in the *Ochotona* Family Driven by the Cold Environmental Stress

**DOI:** 10.1371/journal.pone.0001472

**Published:** 2008-01-23

**Authors:** Jie Yang, Zhen Long Wang, Xin Quan Zhao, De Peng Wang, De Lin Qi, Bao Hong Xu, Yong Hong Ren, Hui Fang Tian

**Affiliations:** 1 Key Laboratory of Qinghai-Tibetan Plateau Biological Evolution and Adaptation, Northwest Institute of Plateau Biology, The Chinese Academy of Sciences, Xining, Qinghai, China; 2 Graduate School of the Chinese Academy of Sciences, Beijing, China; 3 College of Life Science, Qufu Normal University, Qufu, Shandong, China; 4 Microbiology Department, Shijiazhuang Center for Disease Control and Prevention, Shijiazhuang, Hebei, China; New York University College of Dentistry, United States of America

## Abstract

**Background:**

Environmental stress can accelerate the evolutionary rate of specific stress-response proteins and create new functions specialized for different environments, enhancing an organism's fitness to stressful environments. Pikas (order *Lagomorpha*), endemic, non-hibernating mammals in the modern Holarctic Region, live in cold regions at either high altitudes or high latitudes and have a maximum distribution of species diversification confined to the Qinghai-Tibet Plateau. Variations in energy metabolism are remarkable for them living in cold environments. Leptin, an adipocyte-derived hormone, plays important roles in energy homeostasis.

**Methodology/Principal Findings:**

To examine the extent of leptin variations within the *Ochotona* family, we cloned the entire coding sequence of pika leptin from 6 species in two regions (Qinghai-Tibet Plateau and Inner Mongolia steppe in China) and the leptin sequences of plateau pikas (*O. curzonia*) from different altitudes on Qinghai-Tibet Plateau. We carried out both DNA and amino acid sequence analyses in molecular evolution and compared modeled spatial structures. Our results show that positive selection (PS) acts on pika leptin, while nine PS sites located within the functionally significant segment 85-119 of leptin and one unique motif appeared only in pika lineages-the ATP synthase α and β subunit signature site. To reveal the environmental factors affecting sequence evolution of pika leptin, relative rate test was performed in pikas from different altitudes. Stepwise multiple regression shows that temperature is significantly and negatively correlated with the rates of non-synonymous substitution (Ka) and amino acid substitution (Aa), whereas altitude does not significantly affect synonymous substitution (Ks), Ka and Aa.

**Conclusions/Significance:**

Our findings support the viewpoint that adaptive evolution may occur in pika leptin, which may play important roles in pikas' ecological adaptation to extreme environmental stress. We speculate that cold, and probably not hypoxia, may be the primary environmental factor for driving adaptive evolution of pika leptin.

## Introduction

The environment is an important driver for organismic natural selection. Environmental changes or climatic fluctuations can make organisms evolve rapidly into different morphologic or taxonomic groups or create new functions specialized in different individual living environments [1]. Organismic evolution is the process by which an organism must repetitiously overcome new conditions and create a new set of unique metabolic reactions to a particular environmental stress. This adaptive response to changing environmental conditions results in an acceleration of evolutionary rate of the lineage and the functional evolution of specific stress-response proteins, which favors organismic fitness to a new environment. All evolution of phenotypes results from minor increases in mutation rates of genes related to a particular stress [2]–[4]. The field of modern molecular evolution provides powerful tools for us to study the relationships between the functional changes of proteins and the rates of nucleotide and amino acid substitution [5], [6]. Comparisons of the ratio of non-synonymous (Ka)/synonymous (Ks) substitutions (ω = Ka/Ks) have become a useful means for quantifying the impact of natural selection on molecular evolution [7], [8]. Synonymous mutations are generally neutral in the course of evolution and do not result in changes to the amino acids in a protein, while non-synonymous mutations can occur under strong selective pressure and result in the altering of the amino acids in a protein. A value of ω = 1 denotes a neutral mutation, ω less than 1 purifying selection which describes selection against new variants, while ω greater than 1 denotes positive selection (adaptive molecular evolution) in that non-synonymous mutations offer fitness advantages to the protein [9]. A purifying selection may aid in the detection of regions or residues of functional importance. However, much interest in evolution focuses on positive selection because it is associated with adaptation and the evolution of new forms or functions.

Pikas are small non-hibernating, diurnal lagomorphs (rabbits and relatives; order *Lagomorpha*) that belong to the family *Ochotonidae*. Pikas are endemic to the modern Holarctic Region [10], [11]. There are approximately 26 species throughout the world; most are restricted to Asia with only three pika species that presently live outside of Asia. In North America, the only two species (the American pika, *O. princes*, and the collared pika, *O. collaris*) are discontinuously distributed across the mountainous areas of western North America, from the southern Sierra Nevada and Rocky Mountains to central British Columbia. In Europe, the range of the steppe pika (*O. pusilla*) extends west to the Ural Mountains. In Asia, pikas are found throughout central Asia, in the Himalayan massif and associated ranges, and across Western Siberia to Sakhalin Island and onto Hokkaido Island, Japan. Among the species in Asia, approximately 18 species are concentrated in the Qinghai-Tibet Plateau and adjacent areas, accounting for greater than 70% of all pikas in the world [12]–[15]. Obviously, most of the species are confined to those regions with either high altitudes or high latitudes and live in cold climates. The species diversification of pikas on Qinghai-Tibet Plateau implies that pikas are particularly fitted for survival under the environment or climate of the Qinghai-Tibet Plateau. Hypoxia and cold are the two most remarkable climate characteristics of the Qinghai-Tibet Plateau, known to be the highest plateau in the world. Thermoregulation is very important for animals' survival in cold environment. Under these extreme conditions, animals show apparent alteration in energy expenditure in order to meet the varying metabolic requirements imposed by environmental stresses [16]. During evolution, plateau pikas (*O. curzoniae*), the keystone species in the Qinghai-Tibet Plateau ecosystem [17], have become highly hypoxia- and low temperature-tolerant mammals with markedly high resting metabolic rates (RMR), non-shivering thermogenesis (NST), and a high ratio of oxygen utilization to cope with the cold and hypoxic plateau environment [18]–[20]. Furthermore, plateau pikas also show marked seasonal changes in thermogenic capacities with enhanced nonshivering thermogenesis (NST), cytochrome c oxidase activity and increased production of mitochondrial uncoupling protein 1 (UCP1) in brown adipose tissues (BAT) to deal with the cold in winter [21].

Leptin, the product of the *ob* gene, is a 16-kDa cytokine-like hormone primarily secreted by adipose tissue that acts on hypothalamic centers and regulates the energy balance by determining changes in both food intake and energy expenditure [22]–[24]. Physiologically, plasma leptin concentrations reflect adipose tissue deposits [25] and appear to be negatively regulated by fasting [22], protein kinase C [26], androgens [27] and β-adrenergic agonists [28], whereas feeding [22], [29], oestrogens [30], glucocorticoids [30], [31] and insulin act by stimulating both ob gene expression and leptin secretion [32]. Leptin is considered as an important regulator of adaptive thermogenesis in response to environmental temperature or diet. Leptin administration can increase an animal's body temperature, basal metabolic rates (BMR), and nonshivering thermogenesis (NST) which enhance its ability to tolerate cold stress [33]. Leptin increases energy expenditure by increasing uncoupling protein 2 (UCP2) expression in white adipose tissue (WAT), increasing sympathetic activation of UCP1 gene expression in BAT, and upregulating LPL gene expression in brown adipose tissue (BAT) [34], [35]. Cold exposure can reduce leptin expression by directly acting on adipocytes or indirectly acting via the sympathetic nervous system [36], [37], while hypoxia can increase leptin expression [38]. Obviously, regulation of leptin plays an important role in meeting the fluctuating requirements of energy expenditure and energy intake under different physiological states. Due to the extraordinary environment in which pikas live, and their apparent changes in energy metabolism under cold conditions, we hypothesized that leptin, acting as a cold stress-response protein, plays an important role in the pikas' ecological adaptation to the harsh plateau environment. However, the sequence characteristics of leptin specialized in extreme environmental stress, which is very important for us to understand animals' ecological adaptation mechanism, remains unknown. In our previous study, only leptin from the plateau pika was cloned and was identified as exhibiting divergent mRNA expression at different altitudes [39]. Together, these data led us to ask: is pika leptin sensitive to the cold and hypoxic plateau environment; does pika leptin itself functionally evolve under this environmental stress?

To examine the extent of leptin variation within the *Ochotona* family, we carried out both DNA and amino acid sequence analyses in molecular evolution and compared modeled spatial structures. To identify the environmental factor for driving evolution of pika leptin, we compared leptin sequences of pikas from different altitudes. Therefore, our study first explored the relationships between environmental stresses on the Qinghai-Tibet Plateau and molecular evolution of stress-response genes.

## Results

**Figure 1:**
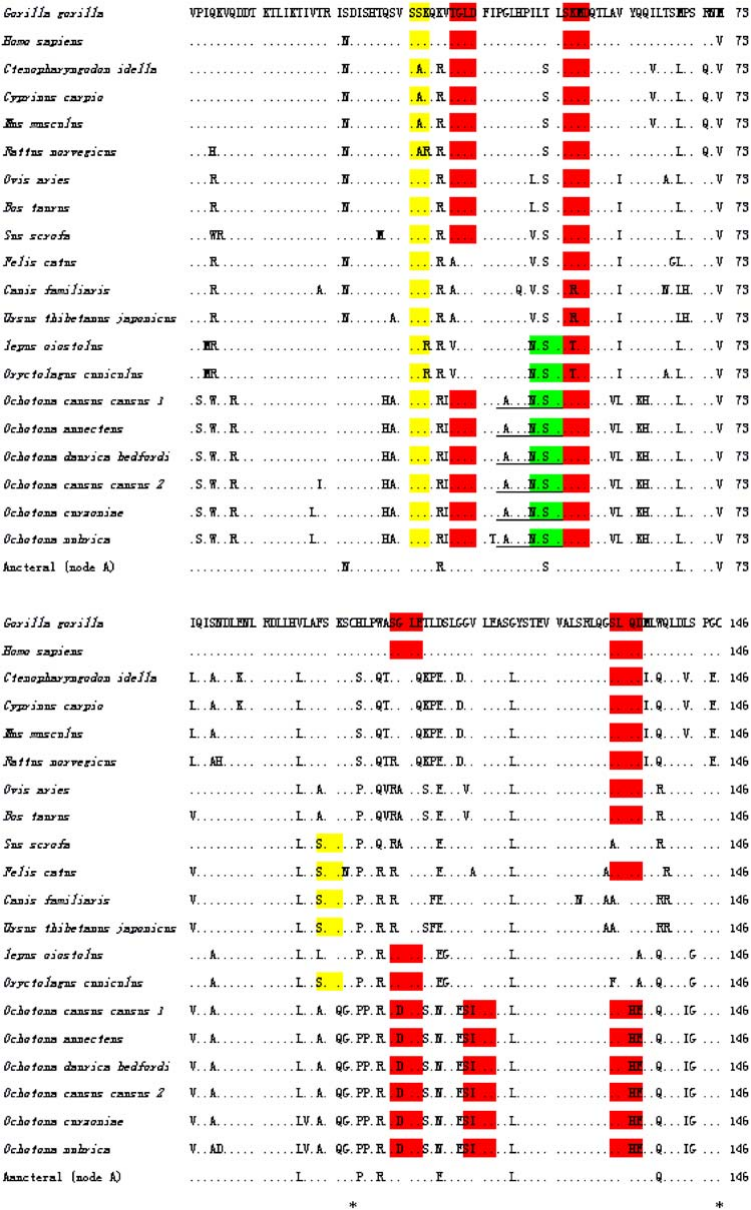
Multiple alignments of leptin amino acids sequences. Residues identical to Gorilla leptin are presented as dots (.). The predicted motifs are shaded by different colors [protein kinase C phosphorylation sites (PKC) are yellow; casein kinase II phosphorylation sites (CK2) are red; N-glycosylation sites are green). Underlined amino acid sequences indicate the motif of the ATP synthase α and β subunit signature site. The numbers at the right are the total numbers of amino acids. Two cysteine residues at 96 and 146 are indicated by asterisks.

A 646-bp fragment in pikas and a 565-bp fragment from both *Lepus oiostolus* and *Oryctolagus cuniculus*, which contained the complete coding region, were cloned, respectively. These sequences were submitted to GenBank and were assigned the following accession numbers: DQ983189 (*Ochotona curzoniae*); EF091861 (*Ochtona nubrica*); EF091863 (*Ochotona cansus cansus* 1); EF091864 (*Ochotona cansus cansus* 2); EF091862 (*Ochotona annecten*); EF091860 (*Ochotona daurica* bedfordi); DQ983190 (*Lepus oiostolus*), and DQ983191 (*Oryctolagus cuniculus*). The deduced amino acid sequences were composed of 167 amino acids and encoded an apparent signal peptide sequence of 21 amino acids with the signal cleavage site between Ala-21 and Val-22. Thus, the mature secreted protein had a predicted molecular weight of 16.086 kDa and a pI of 6.3. To reveal the evolutionary divergence in leptin sequences among lineages, we assembled and analyzed 20 sequences representing samples from different lineages in vertebrates. The result of the multiple alignments for amino acid sequences is shown in Figure 1.

### Evolutionary analysis

#### Phylogenetic tree construction and relative rate analysis

**Figure 2:**
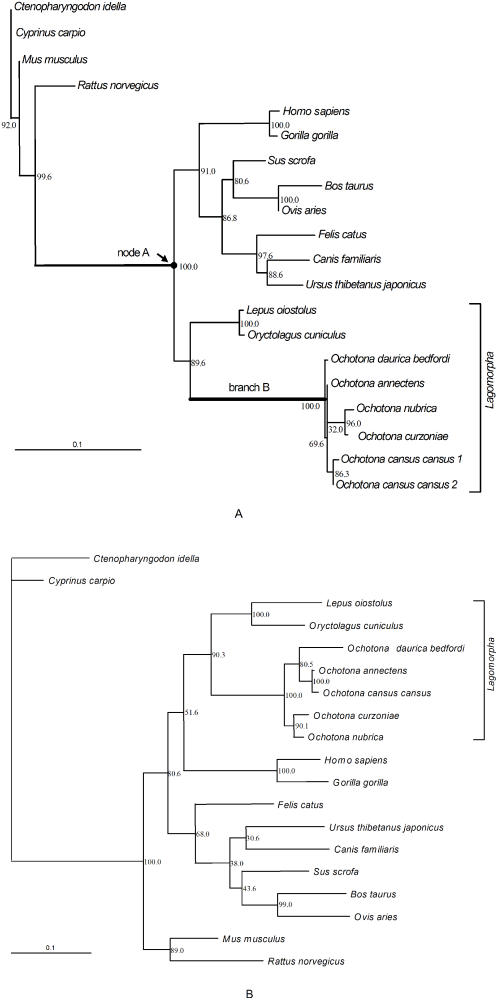
Maximum likelihood tree of the *ob* gene and mitochondrial *cytochrome b* (*cytb*) gene. (A) shows the phylogenetic tree based on nucleotide sequences of *ob* gene. Node A indicates reconstructed ancestral sequences. Branch B indicates the pika branch. (B) shows the phylogenetic tree based on nucleotide sequences of mitochondrial *cytochrome b* (*cytb*) gene. The scale bar of “0.1” means 0.1 nucleotide substitution per site.

The best-fit model of molecular evolution of leptin sequence that was obtained from ModelTest3.7 [40] based on the likelihood ratio test was the HKY+G model. Settings for this model were as follows: base frequencies (A = 0.2162, C = 0.3242, G = 0.2663, and T = 0.1933); transition/transversion ratio (Ti/Tv = 2.3015); and a shape parameter of the gamma distribution of 0.7012. Parameters obtained from this analysis were used for the construction of the phylogenetic trees. All phylogenetic trees constructed by NJ (Neighbor-joining), MP (Maximum parsimony) and ML (Maximum likelihood) methods produced similar topologies; we only show the ML trees constructed from the nucleotide sequences (Figure 2A). The ancestral sequence at node A was reconstructed. To reveal traditional taxonomy among these lineages, phylogenetic trees of mitochondrial *cytochrome b* (*cytb*) gene were constructed with the same methods as the leptin tree. Also, the ML tree of *cytb* gene is shown in Figure 2B. Relative rate test which was used for the estimation of the evolutionary rate among lineages was performed by the pair-wise comparisons of synonymous substitutions (Ks) and non-synonymous substitutions (Ka) for nucleotide and amino acid variations for protein sequences; the node A sequence was used as an outgroup. The results of relative rate test are shown in Tables 1 and 2.

**Table 1:** Individual pair-wise synonymous (Ks) and non-synonymous (Ka) substitutions (*p*-value^a^)

	*Gorilla gorilla*	*Homo sapiens*	*Ovis aries*	*Bos taurus*	*Sus scrofa*	*Canis familiaris*	*Ursus thibetanus japonicus*	*Felis catu*s	*Ochotona cansus 1*	*Ochotona cansus 2*	*Ochotona curzoniae*	*Ochtona nubrica*	*Ochotona annecten*	Ochotona daurica bedfordi	*lepus oiostolus*	*Oryctolagus cuniculus*
*Gorilla gorilla*		0.2178	0.5206	0.1844	0.6731	0.4555	0.1589	0.5087	0.6428	0.7563	0.6237	0.6275	0.7645	0.9015	0.7586	0.3574
*Homo sapiens*	0.1488		0.7439	0.3201	0.4659	0.3073	0.2649	0.7112	0.8494	0.9711	0.8292	0.8360	0.9833	0.8766	0.9886	0.5307
*Ovis aries*	0.8221	0.7893		0.1707	0.2183	0.1524	0.3964	0.9565	0.9259	0.8078	0.9451	0.9361	0.7929	0.6638	0.7865	0.7639
*Bos Taurus*	0.6024	0.9628	0.3867		0.0633	0.0491	0.8381	0.5562	0.5270	0.4341	0.5414	0.5307	0.4188	0.3270	0.4082	0.7982
*Sus scrofa*	0.8929	0.7235	0.8937	0.5781		0.6970	0.0594	0.2707	0.4202	0.5118	0.4071	0.4078	0.5166	0.6307	0.5242	0.2377
*Canis familiaris*	0.3455	0.1530	0.1710	0.0702	0.1891		0.0075	0.0874	0.2599	0.3240	0.2459	0.2464	0.3229	0.4089	0.3460	0.1446
*Ursus thibetanus japonicus*	0.5737	0.2899	0.3191	0.1370	0.3823	0.3466		0.3733	0.4144	0.3330	0.4272	0.4167	0.3193	0.2423	0.3017	0.6666
*Felis catu*s	0.7104	0.3843	0.4934	0.2714	0.5623	0.3802	0.7541		0.8920	0.7766	0.9090	0.8993	0.7620	0.6369	0.7647	0.8063
*Ochotona cansus 1*	0.0252	0.0099	0.0109	0.0040	0.0124	0.1601	0.0650	0.0537		0.3105	0.9392	0.9615	0.5191	0.2589	0.8400	0.6574
*Ochotona cansus 2*	0.0174	0.0067	0.0073	0.0026	0.0083	0.1201	0.0461	0.0382	0.3128		0.5428	0.5154	0.9414	0.4681	0.9790	0.5367
*Ochotona curzoniae*	0.0140	0.0052	0.0056	0.0019	0.0064	0.1034	0.0380	0.0316	0.1450	0.7725		0.9375	0.3388	0.1588	0.8154	0.6728
*Ochtona nubrica*	0.0064	0.0023	0.0024	0.0008	0.0028	0.0548	0.0181	0.0152	0.0422	0.2113	0.1408		0.2215	0.1073	0.8232	0.6604
*Ochotona annecten*	0.0252	0.0099	0.0109	0.0040	0.0124	0.1601	0.0650	0.0537	no	0.3128	0.1450	0.0422		0.2752	0.9932	0.5146
*Ochotona daurica bedfordi*	0.0172	0.0066	0.0072	0.0025	0.0082	0.1188	0.4546	0.0378	0.2949	0.9786	0.7936	0.2186	0.2949		0.8443	0.3944
*lepus oiostolus*	0.9052	0.5643	0.7281	0.5205	0.7927	0.3760	0.6319	0.7972	0.0193	0.0130	0.0101	0.0044	0.0193	0.0128		0.1917
*Oryctolagus cuniculus*	0.5063	0.2767	0.3199	0.2257	0.3873	0.7688	0.8934	0.7409	0.0713	0.0503	0.0413	0.0193	0.0713	0.0496	0.1499	

Note: Ks above diagonal, Ka below diagonal.

*p*-value^a^: exact significant statistical test probability.

**Table 2:** Individual pair-wise comparisons of amino acid variations (*p*-value^a^)

	*Lepus oiostolus*	*Oryctolagus cuniculus*	*Gorilla gorilla*	*Homo sapiens*	*Ovis aries*	*Bos Taurus*	*Sus scrofa*	*Canis familiaris*	*Ursus thibetanus japonicus*	*Felis catus*	*Ochotona daurica bedfordi*	*Ochotona cansus 1*	*Ochotona annecten*	*Ochotona curzoniae*	*Ochtona nubrica*	*Ochotona cansus 2*
*Lepus oiostolus*																
*Oryctolagus cuniculus*	0.2038															
*Gorilla gorilla*	0.6463	0.3789														
*Homo sapiens*	0.3610	0.1891	0.1255													
*Ovis aries*	0.4587	0.2156	0.8152	0.8063												
*Bos taurus*	0.3244	0.1593	0.6409	no	0.4435											
*Sus scrofa*	0.7987	0.4425	0.8048	0.4605	0.5140	0.3289										
*Canis familiaris*	0.2563	0.4836	0.1537	0.0580	0.0470	0.0220	0.1125									
*Ursus thibetanus japonicus*	0.6356	no	0.3987	0.1891	0.1763	0.0833	0.3991	0.1746								
*Felis catus*	0.8033	0.8076	0.4905	0.2297	0.2798	0.1493	0.5746	0.2036	0.7283							
*Ochotona daurica bedfordi*	0.0109	0.0358	0.0087	0.0031	0.0027	0.0010	0.0092	0.1756	0.0450	0.0269						
*Ochotona cansus 1*	0.0169	0.0527	0.0131	0.0048	0.0042	0.0017	0.0141	0.2306	0.0646	0.0396	0.2475					
*Ochotona annecten*	0.0169	0.0527	0.0131	0.0048	0.0042	0.0017	0.0141	0.2306	0.0646	0.0396	0.2475	no				
*Ochotona curzoniae*	0.0070	0.0240	0.0057	0.0020	0.0017	0.0006	0.0060	0.1319	0.0309	0.0181	0.5026	0.1022	0.1022			
*Ochtona nubrica*	0.0028	0.0105	0.0024	0.0083	0.0065	0.0002	0.0024	0.0716	0.0142	0.0079	0.1193	0.0210	0.0210	0.0992		
*Ochotona cansus 2*	0.0109	0.0358	0.0087	0.0031	0.0027	0.0010	0.0092	0.1700	0.0450	0.0269	no	0.2475	0.2475	0.5026	0.1193	

*p*-value^a^: exact significant statistical test probability

#### Selective pressure analysis

To analyze the possibility that positive selection acts on pika leptin, we used the maximum-likelihood codon model from the CODEML program in the PAML package [41]. The topology of the ML tree mentioned above was modified for all CODEML analyses. We treated branch B as the foreground branch and all other branches in the phylogeny as background branches (Figure 2A). Likelihood values and parameters, as well as likelihood ratio test statistics, are shown in Tables 3 and 4. In the branch-specific likelihood analysis, the LRT statistic for the comparison of the one-ratio model vs. the two-ratio model was 2Δℓ = 6.69266 with *p* = 0.009681 and df = 1. Therefore, the ω ratio for branch B (ω_1_ = 0.5360) was significantly different from that for all other branches (ω_0_ = 0.1767). To test whether ω_B_ was significantly higher than 1, the log likelihood value was calculated under the two-ratio model but with ω_B_ = 1 fixed, yielding the log likelihood value of -1366.409742. The two-ratio model that did not place the constraints on ω_B_ (Table 4) was not significantly better as the test statistic was 2Δℓ = 2.94 with *p* = 0.086 and df = 1. Therefore, ω_B_ was not significantly greater than 1 at the 5% significance level. In site-specific likelihood models, M2a (positive selection) did not detect the existence of positive selection sites and had the same log likelihood value as M1a. Thus, the LRT statistic of the M1a-M2a comparison was not of statistical significance. The discrete model (M3) with K = 3 site classes suggested that 2.95% of sites were under positive selection with ω_2_ = 1.25333 and identified one amino acid (92F) under positive selection at the 89.0% probability. The LRT statistic of M3-M0 comparison was 2Δℓ = 13.051004 with *p* = 0.001466 and df = 2, so the M3 was significantly better than M0. M8 (beta & ω), suggested that 1.767% of sites were under positive selection with ω = 1.22961 and identified one site under positive selection (the same as under M3). The differences between M7 and M8 were not statistically significant, as 2Δℓ = 0.16274 with *p* = 0.9219 and df = 2. In the branch-site models, model A identified 14 sites under positive selection. The LRT test statistic of comparison of Model A vs. M1a was 2Δℓ = 4.922874 with *p* = 0.08531 and df = 2, therefore, model A did not fit the data significantly better than did model M1a. Model B suggested 29.348% of sites were under positive selection with ω = 1.31007 and the identification of 20 sites under positive selection. The comparison of model B vs. M3 yielded 2Δℓ = 3.015114 with *p* = 0.2215 and df = 2. Because both models allowed for positive selection, this comparison was not of biological significance.

**Table 3:** Likelihood values and parameter estimates for the leptin gene

Model code	estimate of parameters	ℓ	positively selected sites
M0: one-ratio	ω = 0.2125	−1369.756072	None
Branch-specific model			
Two-ratio	ω_0_ = 0.1767, ω_1_ = 0.5360	−1366.409742	
Site-specific models			
M1a: nearly neutral (K = 2)	*p*_0_ = 0.90444, (*p*_1_ = 0.09556)	−1365.192539	Not allowed
M2a: positive selection (K = 3)	*p*_0_ = 0.9044, *p*_1_ = 0.04840 (*p*_2_ = 0.04716) ω_2_ = 1.0000	−1365.192539	None
M3: discrete (K = 3)	*p*_0_ = 0.32356, *p*_1_ = 0.64693 (*p*_2_ = 0.02951) ω_0_ = 0.00860, ω_1_ = 0.29657, ω_2_ = 1.25333	−1363.230570	92F (*p* = 0.890)
M7: beta	*p* = 0.73208, q = 2.40834	−1363.617625	None
M8: beta&ω	*p*_0_ = 0.98233, *p* = 0.88393, q = 3.20774 (*p*_1_ = 0.01767), ω = 1.22961	−1363.536255	92F (*p* = 0.571)
Branch-site models			
Model A	*p*_0_ = 0.55665, *p*_1_ = 0.05478 (*p*_2_+*p*_3_ = 0.38857), ω_2_ = 1.0	−1362.731102	2P 60V 63Q 95S 103G 106T (at 0.5<*p*<0.6); 7Q 28Q 29S 44G 62Q 94K 98L (at 0.6<*p*<0.7); 108D (at *p*>0.8)
Model B	*p*_0_ = 0.47459, *p*_1_ = 0.23194 (*p*_2_+*p*_3_ = 0.29348), ω_0_ = 0.07001, ω_1_ = 0.45133, ω_2_ = 1.31007,ω_3_ = 1.31007	−1361.723013	4Q 95S 103G 106T 111G 113V (at 0.5<*p*<0.6); 29S 59A 60V (at 0.6<*p*<0.7); 2P 7Q 28Q 36V 44G 62Q 63Q 92F 94K 98L (at 0.7<*p*<0.8); 108D (at *p*>0.8)

**Table 4:** Likelihood ratio test statistics (2Δℓ) for leptin

	2Δℓ	df	*p*-value
LRT of ω at branch B (Fig. 2)			
one ratio vs. two ratio	6.69266	1	0.009681
LRTs of variable ω values among sites			
M1a vs. M2a	0	2	1
M7 vs. M8	0.16274	2	0.9219
one ratio vs. M3	13.051004	2	0.001466
LRTs of variable ω values along branch B (Fig. 2)			
M1a vs. Model A	4.922874	2	0.08531
M3 vs. Model B	3.015114	2	0.2215

#### Secondary and tertiary structure analysis

To be consistent with the evolutionary analysis, we only analyzed mature protein (146 amino acids) without the signal peptide sequence. The consensus methods of secondary structure prediction suggested that pika leptins, like those of all other lineages, were composed of 4 helixes with two conservative CYS sites at 96 and 146, forming one disulfide bond for structural stabilization. The tertiary structure of pika leptin was based on a model of human leptin (1ax8_) [42] from the Protein Data Bank and is shown in Figure S1, which is published as supporting information on the PLoS One web site. The motifs predicted in the leptin structure indicated that the protein kinase C phosphorylation sites (PKC) and the casein kinase II phosphorylation sites (CK2) are conserved among these lineages; a N-glycosylation site existed only in pika leptin and rabbit leptin; and a unique motif existing only in pika leptin was a single ATP synthase α and β subunit signature site (Figure 1).

#### Analyses of leptin sequences of plateau pikas from different altitudes

**Figure 3:**
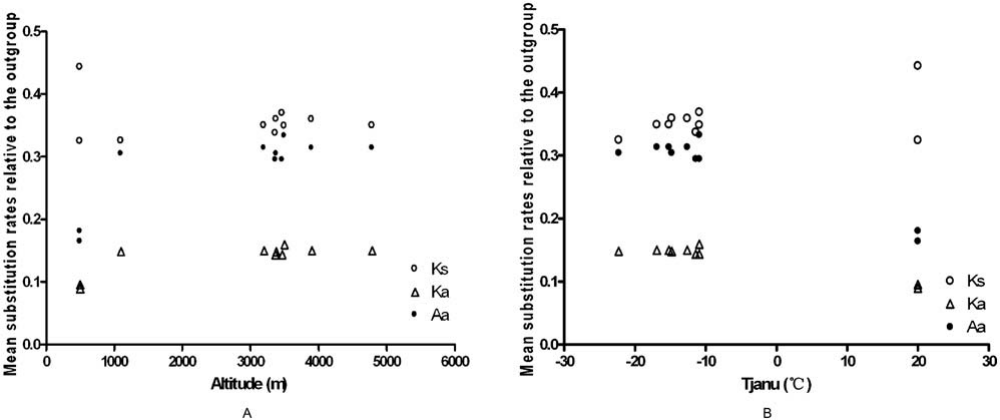
Relationship between mean rates of synonymous substitution (Ks), non-synonymous substitution (Ka) and amino acid substitution (Aa) relative to outgroup and altitude (in m) and mean January actual temperature (Tjanu, °C). (A) shows the relationship between mean rates of Ks, Ka and Aa relative to outgroup and altitude (in m). (B) shows the relationship between mean rates of Ks, Ka and Aa relative to outgroup and mean January actual temperature ((Tjanu, °C).

To reveal the major environmental factors affecting sequence evolution of pika leptin, relative rate test was performed in pikas from different altitudes. Human leptin sequence was used as an outgroup. Stepwise multiple regression analysis was used to determine how mean January actual temperature (Tjanu, °C) and altitude (Al, in m) influenced mean rates of synonymous substitution (Ks), non-synonymous substitution (Ka) and amino acid substitution (Aa). The results showed that Tjanu was significantly and negatively correlated with Ka (R^2^ = 0.91, F = 81.33, df = 1, 8, P<0.001) and Aa (R^2^ = 0.90, F = 75.11, df = 1, 8, P<0.001), whereas Al is not significantly correlated with Ks, Ka and Aa. (Table 5, Figure 3). Thus, comparative to rat and rabbit, pikas with relatively lower mean January actual temperature reached a relatively higher substitution rate of Ka and Aa. Altitude was not included in the model and, thus, did not significantly affect the substitution rates of Ks, Ka and Aa of pika leptin.

**Table 5:** Models obtained by means of stepwise multiple regression analysis on factors that explained the variation of non-synonymous substitutions (Ka) and amino acid substitutions (Aa) (dependent variables) of pikas, rat and rabbit.

Dependent Variable	β	B±SE	t	*P*
non-synonymous substitutions				
Intercept	−0.954	0.126±0.00	45.601	<0.001
Tjanu		−0.002±0.00	−9.018	<0.001
amino acid				
Intercept		0.253±0.01	36.595	<0.001
Tjanu	−0.951	−0.004±0.00	−8.667	<0.001

The independent variables were mean January actual temperature (Tjanu, °C) and altitude (Al, in m). Standardized (β) and nonstandardized (*B*) regression coefficients and their standard errors are shown. The table provides results from *t*-tests (*t*) and associated probability (*P*) levels.

Note: The survival environment of the standard trial animal, rat and rabbit, was according to the feeding conditions in laboratory (altitude = 500 m, temperature = 20°C).

## Discussion

In the present study, we have compared the entire coding sequence of leptin from different lineages of representative mammals in order to help us identify the variation of functional sites and to understand the mechanism of functional evolution of pika leptin. The phylogenetic tree of leptin yielded similar topology to that of the mitochondrial *cytb* gene, and thus, was consistent with traditional morphological assortment. However, comparing divergent distance with the inner branch length in the phylogenetic tree of leptin, we found the pika branch (shown as branch B in Figure 2A) to be significantly longer than any of the other lineage branches, implying that the variation of leptin sequence in the pika lineage is great. Next, to identify the evolutionary rate of leptin to be a result of neutral evolution or natural selection, the relative rate test was performed. The results showed that pika branch was significantly different (P<0.05) from all other lineages in nonsynonymous (Ka) and amino acid variation, but not in synonymous (Ks). Interestingly, in comparison of evolutionary rate between pika and rabbit lineages we found that the differences of the evolutionary rate between these two lineages were significant, but the differences between rabbit and all other lineages were not significant. In traditional taxonomy, pika and rabbit have a closer kinship than any other compared lineages and belong to the same family (*Lagomorpha*). However, for leptin evolution, the taxonomic relationship between these two lineages is changed and the divergence between pika and rabbit lineages appears to be significantly greater than that between rabbit and all other lineages. That is to say, the evolutionary rate of leptin between rabbit and other selected lineages was stable and conformed to the molecular clock model. While for pika, after diverging with rabbit, the evolutionary rate of pika leptin may be accelerated.

To determine the nature of variation sites occurring in pika leptin, a set of evolutionary analysis was performed. A comparison of the one-ratio vs. the two-ratio in branch-specific models revealed the ω ratio along the pika lineage was significantly different from all other lineages. In site-specific models, both the M8 (beta & ω) and M3 (discrete) models demonstrated unconsentaneous ω ratios among sites, yielding a ω ratio of 1.22961 and 1.25333, respectively, and predicted one common site under positive selection, 92F. Under branch-site models, model B provided a ω ratio of 1.31007 and identified the following sites to be under positive selection: 2S, 4W, 7R, 28H, 29A, 36I, 44A, 59V, 60L, 62K, 63H, 92A, 94Q, 95G, 98P, 103D, 106S, 108N, 111E, 113I. The ancestral sequence reconstructed by the models of Goldman and Yang [43] and of Yang, Kumar, and Nei [44] suggested the following amino acid changes along the pika branch: 2SP, 4WQ, 7RQ, 28HQ, 29AS, 36IV, 44AG, 59VA, 60LV, 62KQ, 63HQ, 94QK, 95GS, 98PL, 103DG, 108NE, 113IV, and 134HQ. Obviously, sites under positive selection, inferred by Bayes prediction, were highly consistent with those from the reconstruction of the ancestral sequence. Therefore, our evolutionary analysis confirmed the previous hypothesis that adaptive evolution occurred in pika leptin.

The establishment of new or modified function of a protein under specific stress is derived from the adaptive evolution in this protein. We speculate the possible effect of positive selection sites on the functional evolution of pika leptin interpreted from the analysis of literature on the evolutionary, functional-structural, and biochemical information concerning the leptin protein. Previous investigations indicated that segment 85-119 in leptin protein was of special functional significance and underlied the functional differences between human and other non-hominoid leptins [45]–[48]; the key binding sites between leptin and its receptor were 9D, 12T, 15K, 16T, 82N, 85D, and 86L; the important sites for leptin signaling activation were 20R 29S, 30V, 31S, 34Q, 35R 41F, 75Q 115E, 117S, 120S, 121T, 122E 138Q, 139Q, and 142V. Mutations at these sites showed strongly decreased binding affinity or signaling activation for its receptor [49]–[52]. In pika leptin, positive selection (PS) mutations occur at the following 20 sites: P2S, Q4W, Q7R, Q28H, S29A, V36I, G44A, A59V, V60L, Q62K, Q63H, F92A, K94Q, S95G, L98P, G103D, T106S, D108N, G111E, and V113I. Nine PS sites locate within the functionally significant segment 85-119 of leptin: F92A, K94Q, S95G, L98P, G103D, T106S, D108N, G111E, and V113I, implying an important conclusion that the function of pika leptin may be divergent from the other leptins in all compared lineages. Additionally, the G44A mutation results in a unique motif appearing only in the pika lineage, the ATP synthase α and β subunit signature site, composing of the functional sites (nucleotide-binding site for ATP and ADP in the α subunit and catalytic activity in the β subunit) of the ATP synthase complex that take part in energy transduction in living cells [53]. This motif predicted in pika leptin appears to be consistent with its general role in energy regulation and also seems to be associated with the requirement of varying energy expenditure under extreme environmental stress. The exact experimental evidence for the existence and function of the ATP synthase α and β subunit signature site in pika leptin needs to be further studied. Substitution by Ala at site 29 occurs in a key site for signaling, changing from a polar (Ser) to a non-polar (Ala) residue. What effect this substitution has on receptor signal activation requires further study. Taken together, these analyses mentioned above led us to suppose that for pika leptin, nine sites under positive selection were within the functionally significant segment 85-119 and one unique motif (ATP synthase α and β subunit signature site) appeared only in the pika lineage, indicating the functional variation of pika leptin.

It is known that cold and hypoxia are the two most remarkable climatic characteristics of the Qinghai-Tibet Plateau. To identify the environmental factor driving the adaptive functional variation of pika leptin, we collected pikas from different altitudes, five species from the Qinghai-Tibet Plateau (average altitude >3000 m) and the other from the Inner Mongolia steppe (altitude of 1300 m). We also collected plateau pikas from three altitudes (3200 m, 3900 m and 4790 m). Because most of the literature concerns cold survival environment in pika species, it is difficult to find a pika species living in a warmer climate. We therefore used trial animals, rabbit and rat as controls living under the environment of warmer temperature and lower altitude. Mean rates of synonymous substitution (Ks), non-synonymous substitution (Ka) and amino acid substitution (Aa) relative to the outgroup-human were investigated and stepwise multiple regression was used to determine how mean January actual temperature (Tjanu, °C) and altitude (Al, in m) affected these substitution rates. The results of stepwise multiple regression showed that pikas with relatively lower mean January actual temperature reached relatively higher substitution rates of Ka and Aa, while altitude was not included in the model, and thus, did not significantly affect the substitution rates of Ks, Ka and Aa. Multiple alignment of sequences of plateau pika leptin from different altitudes also showed that there was only one synonymous mutation in the nucleotide sequences, but no changes in amino acid sequences. The relation between altitude and barometric pressure or inspired oxygen pressure is negatively correlated [54], [55]. Namely, as the altitude increases, the oxygen content of the air decreases, and thus hypoxia aggravates. Therefore, hypoxia differs at the different altitudes. In addition, the evolutionary history of the pika family is also indicative of the cold adaptation in the *Ochotona* family. Fossil records show that this family had an Asiatic origin with the earliest emergence of pikas hypothesized to be during the late Miocene in central Asia and migrated to North America through the Bering Strait during the Mid-Pliocene [56], [57]. Time of divergence within the *Ochotona* family was consistent within the historical episodes of both geologic and climatic changes. The frequent uplift of the Qinghai-Tibet Plateau from 3.4–1.6 mya [58], [59] resulted in strong environmental changes (climatic fluctuations), potentially producing strong selective pressures that resulted in varied clades of *Ochotona* of the Qinghai-Tibet Plateau. With gradual global warming, there were apparent distribution trends toward increased elevations and contracted ranges in pika populations, eventually resulting in the global extinction of some species [13]. Obviously, pikas have been living in a harshly cold environment both in ancient and present time. Together, all these data indicate that cold, and probably not hypoxia, may be the primary environmental factor for driving adaptive evolution of pika leptin. Furthermore, similar changes of pika leptin in the two regions (Qinghai-Tibet Plateau and Inner Mongolia steppe) lead us to suppose that the adaptive evolutionary variation of leptin may have occurred within the entire *Ochotona* family throughout the world due to their common evolutionary history of Asiatic origin and their similar cold environment for survival in all ages.

Adaptive thermogenesis is the main way of heat production for small mammals in response to cold environmental stress and is produced mainly by means of nonshivering thermogenesis (NST) associated with an increase in BAT weights, mitochondria protein concentrations, and uncoupling protein 1 (UCP1) mRNA expression [60], [61]. Leptin administration increases an animal's body temperature, basal metabolic rates (BMR), NST, and UCP1 mRNA expression in BAT [33]–[35]. Compared with other small mammals that live in warmer environments, pikas generally show markedly high levels of resting metabolic rates (RMR) and non-shivering thermogenesis (NST) to cope with the harsh plateau environment [20]. Therefore, we speculate that adaptive functional evolution in pika leptin may play an important role in contributing to fitness enhancement for pikas' survival in a stressful environment. Leptin also regulates lipid and glucose metabolism and insulin action [62], [63]. Leptin stimulates fatty acid oxidation and glucose uptake in skeletal muscle [64]–[67], and inhibits glucose output, lipogenesis in liver [68], [69], and insulin secretion [70], which increase insulin sensitivity. Leptin can regulate osteoblastic osteocalcin production via insulin response. Bromage speculates that natural selection may be acting on insulin production, achieving this by altering leptin in such a way as to enhance hypothalamic sensitivity to leptin and the resultant osteocalcin production (Bromage, pers comm.). These facts mentioned above make it plausible that adaptive evolution in pika leptin may be targets of study in order to help us to clarify the adaptive mechanism for small mammals living under extremely stressful environments, and to identify potential therapeutic strategies for human's disease associated with metabolic disorders.

In summary, our study confirmed the previous hypothesis that leptin is a cold stress-response protein and that cold probably is the primary environmental factor for driving the adaptive functional evolution of leptin within the native cold-adapted *Ochotona* family, contributing to fitness enhancement for the pikas' survival in a stressful environment. We also put forward an important hypothesis: adaptive functional evolution of pika leptin may be a common characteristic of the entire *Ochotona* family throughout the world.

## Materials and Methods

### Sampling location

Geographical and climatological data for pikas collected in this study were shown in Table 6. We also collected rabbit (*Oryctolagus cuniculus*) and hare (*Lepus oiostolus)* as comparison with pikas. The identification of pika species was performed by sequencing the mitochondrial *cytochrome b* (*cytb*) gene according to Yu et al [71].

**Table 6:** Geographical and climatological data for pikas in this study

Species	location	Habitat	Lat	Long	Al	Tm	Tjanu	Tjuly	Ffp	Rn
*Ochotona daurica bedfordi*	Inner Mongolia Grassland Station^a^	steppe	43°38′N	116°42′E	1100	−0.4	−22.3	18.7	110	350.0
*Ochotona nubrica*,	Jiuzhi county, Qinghai	rock shrub	33°27′N	101°29′E	3600	0.2	−10.9	9.8	38	764.1
*Ochotona cansus cansus 1*	Jiuzhi county, Qinghai	shrub	33°27′N	101°55′E	3470	0.2	−10.9	9.8	38	764.1
*Ochotona cansus cansus 2*	Saierlong, Henan county, Qinghai	shrub	34°31′N	102°01′E	3380	−2.4	−14.8	8.6	12	460.2
*Ochotona annecten*	Senduo, Guinan county, Qinghai	Alpine meadow	35°30′N	101°06′E	3370	2.1	−11.4	13.4	90	403.1
*Ochotona curzoniae*	Haibei Station^b^	Alpine Meadow	37°29′N	101°12′E	3200	−1.7	−15.2	9.8	20	582.1
*Ochotona curzoniae*	Dawu, Guoluo county, Qinghai	alpine meadow	37°25′N	100°30′E	3900	−0.6	−12.6	9.7	23	573.2
*Ochotona curzoniae*	Montain KunLun, Qinghai	alpine meadow	35°42′N	94°04′E	4790	−5.6	−16.9	5.3	10	262.2

Note: Symbols of variables are as follows: Al = altitude (in m); Lat = latitude; Long = longitude; Tm = mean annual temperature(°C); Tjanu = mean January actual temperature(°C); Tjuly = mean July actual temperature (°C); Ffp = frost-free period (days); Rn = mean annual rainfall (in mm). Climatic data were obtained from local weather bureau.

Inner Mongolia Grassland Station^a^ = Inner Mongolia Grassland Ecosystem Research Station, the Chinese Academy of Sciences; Haibei Station^b^ = Haibei Research Station of the Alpine Meadow Ecosystem, the Chinese Academy of Sciences.

The identification of pika species was performed by sequenceing the mitochondrial *cytochrome b* gene according to Yu et al [71]. The sequences of the mitochondrial *cytochrome b* gene in pikas were compared with those in the GenBank database.

### Cloning of leptin cDNA

Total RNA from white adipose tissue was isolated using the TRIzol Reagent (Invitrogen, USA) and treated with RNase-free DNase I (TaKaRa Biotechnology Co. Ltd). RT-PCR was performed using the Access RT-PCR System (Promega, USA). All of the above procedures were done according to the corresponding manufacturer's instructions. The target DNA fragments of expected sizes were purified and subcloned into the pGEM-T Easy Vector (Promega, USA) and then sequenced. We used the following primer pairs for pika leptin amplification: PKLEPFOR (forward primer: 5′-aggaaggaaaatgcggtg-3′) and PKLEPREV (reverse primer: 5′- tggaggagtaaaagagaaatgg-3′). The primer pairs of RBLEPFOR (forward primer: 5′-aggaaggaaaatgcggtg-3′) and RBLEPREV (reverse primer: 5′-gctttggaagggcttggag-3′) were used for rabbit leptin.

For phylogenetic and evolutionary analyses, we used additional published sequences of leptin and mitochondrial *cytochrome b* (*cytb*) gene for the lineages shown in Table S1, which is published as supporting information on the PLoS One web site.

### Sequence analysis

The nucleotide and deduced amino acid sequences were compared with the sequences in the GenBank database using the BLAST program (http://www.ncbi.nlm.nih.gov). The signal peptide was predicted using the SignalP tool (http://www.cbs.dtu.dk/services/SignalP). Multiple alignments were done using the program CLUSTALX 1.81 [72]. The functional amino acid motifs were predicted using the MotifScan program in the PROSITE database of protein families and domains (http://www.expasy.org/prosite). The secondary sequence structure was predicted using the consensus methods of Sspro, Sspro8 [73], ACCpro, CONpro [74], CMAPpro, and CCMAPprothe [73] on the SCRATCH server (http://www.igb.uci.edu/tools/scratch/).

Tertiary structures were modeled using both automated and alignment modes of homology modeling provided by the SWISS-MOELD Server (http://swissmodel.expasy.org) with the reference template of *Homo sapiens* leptin (PDB ID code: 1ax8_) [42]. For visualization and manipulation of the 3D molecule, we used the spdbv 3.7 tool (http://swissmodel.expasy.org/spdbv/) [75].

### Evolutionary analysis

Phylogenetic trees were constructed using three different tree-making algorithms, neighbor-joining (NJ), maximum likelihood (ML), and maximum parsimony (MP), in version 3.66 of the PHYLIP software package using both nucleotide and amino acid sequences, respectively [76]. The stability among the clades of the phylogenetic tree was assessed by taking 1000 replicates of the dataset and performing analyses using the following programs: SEQBOOT, DNADIST, FITCH, DNAML, DNAPARS, PRODIST, PROTPARS, PROML, and CONSENSE from the PHYLIP software package. Common carp and grass carp were used as outgroups for all trees. Relative rate tests were performed using the program RRTree version 1.1 (http://pbil.univ-lyonl.fr/software/rrtree.html) [77]. The ModelTest 3.7 [40] and PAUP* 4.0b10 [78] software were used to determine the best-fit model of molecular evolution and to compute the parameters of base frequencies, transition/transversion rate ratios (Ti/Tv), and gamma distribution shape parameters for the construction of phylogenetic trees and analyses of codon maximum likelihood.

### Selective pressure analysis

Analyses were performed using the CODEML program from PAML version 3.15 [41]. For a given tree and codon model, CODEML finds the set of parameter values (i.e., the likelihood score). Nested models were compared using a likelihood ratio test (LRT) [9]. The LRT statistic was calculated as twice the difference in maximum likelihood values (2Δℓ) between nested models. The significance of the LRT statistic was determined using a Χ^2^ distribution. Because a very high divergence can reduce the power for the detection of positive selection under models of variable ω ratios among sites [79], we excluded the sequence of fish and rodents, leaving other sequences in the dataset. To examine the selective pressure acting on the pika leptin gene, three codon substitution models of maximum likelihood analysis were performed: branch-specific likelihood models, site-specific likelihood models, and branch-site likelihood models. The branch-specific models allow for variable ω ratios among branches but invariable ω ratios in sites in the tree and can be implemented for the study of changes in selective pressures in specific lineages [41], [79]. The null model assumed the same ω ratio for all lineages in the tree (one-ratio model) and the two-ratio models assigned two ω ratios for the foreground (ω_1_) and background branches (ω_0_). The site-specific model allows the ω ratio to vary among sites but fix one ω ratio in all lineages [80]. Three pairs of models, M1a (Nearly Neutral) vs. M2a (Positive Selection), M7 (beta) vs. M8 (beta & ω), and M0 (one-ratio) vs. M3 (discrete), were carried out in site-specific models [81]. The branch-site models (models A and B) allow the ω ratio to vary both among sites and among lineages and were used to detect positive selection that affects only a few sites along a few lineages [79]. In model A, ω_0_ was assigned 0<ω_0_<1, and ω_1_ was fixed at 1; hence, positive selection was permitted only in the foreground branch [82]. In model B, ω_0_ and ω_1_ are free and, thus, some sites may evolve by positive selection across the entire phylogeny, whereas other sites may evolve by positive selection in just the foreground branch. Model A is compared with M1a (Nearly Neutral) and model B is compared with M3 (discrete). Positive selection is indicated when a freely estimated ω parameter is greater than 1 and the LRT reaches a statistically significant level. We applied ML reconstruction of the ancestral sequence using the models of Goldman and Yang [43] and of Yang, Kumar, and Nei [44]. The Bayes theorem was used to identify candidate positive selection sites [83].

Stepwise multiple regression analysis was used to determine how mean January actual temperature (Tjanu, °C) and altitude (Al, in m) influenced mean rates of synonymous substitution (Ks), non-synonymous substitution (Ka) and amino acid substitution (Aa) relative to outgroup.

All procedures involved in the handling and care of animals were in accordance with the China Practice for the Care and Use of Laboratory animals and were approved by China Zoological Society.

## Supporting Information

Figure S1 The modeled tertiary structure of pika leptin with the reference template of Homo sapiens leptin (PDB ID code: 1ax8_). Purple segment of the A-B loop indicates the predicted motif of the ATP synthase α and β subunit signature site. (A) shows all key sites discussed in this article. Blue on the molecular backbone indicates binding sites with the leptin receptor. Yellow indicates the signal sites for activating the leptin receptor. Green denotes positive selection sites. (B) shows only binding sites. Residues and corresponding locations were labeled on the figure. Yellow sites indicate key binding sites for the receptor. Blue indicates minor function in binding with the receptor. Red denotes the key sites both in binding and signaling with the receptor. (C) shows only signaling sites for activating the receptor. (D) shows only positive selection sites occurring in pika leptin.
(6.96 MB TIF)

Table S1 GenBank accession numbers of ob gene and mitochondrial cytochrome b gene of different lineages cited in this study
(0.04 MB DOC)

## References

[1] Huntley B, Webb T (1989). Migration: Species' response to climatic variations caused by changes in the earth's orbit.. J Biogeogr.

[2] Nevo E (2001). Evolution of genome-phenome diversity under environmental stress.. Proc Natl Acad Sci U S A.

[3] Wright BE (1997). Does selective gene activation direct evolution?. FEBS Lett.

[4] Wright BE (2004). Stress-directed adaptive mutations and evolution.. Mol Microbiol.

[5] Nielsen R (2005). Molecular signatures of natural selection.. Annu Rev Genet.

[6] Nei M (2005). Selectionism and neutralism in molecular evolution.. Mol Biol Evol.

[7] Kimura M (1983). The Neutral Theory of Molecular Evolution..

[8] Ohta T (1992). The nearly neutral theory of molecular evolution.. Annu Rev Ecol Syst.

[9] Yang ZH, Nielsen R, Goldman N, Pedersen AM (2000). Codon-substitution models for heterogeneous selection pressure at amino acid sites.. Genetics.

[10] Corbet GB (1978). The Mammals of the Palaearctic Region: A Taxonomic Review..

[11] Hoffmann RS, Wilson DE, Reeder DM (1993). Order Lagomorpha.. Mammalian Species of the World, A Taxonomic and Geographic Reference.

[12] Feng ZJ, Zheng CL (1985). Studies on the pikas (genus Ochotona) of China-Taxonomic notes and distribution.. Acta Therio Sinica.

[13] Grayson DK (2005). A brief history of Great Basin Pikas.. J Biogeogr.

[14] Hall ER (1981). The Mammals of North America 2^nd^ ed..

[15] Smith AT, Formozov NA, Hoffman RS, Zheng CL, Erbajeva MA, Chapman JA, Flus JEC (1990). The pikas.. Rabbits, hares and Pikas: Status survey and conservation action plan.

[16] Storey KB, Storey KB (1999). Stress-induced gene expression in freeze tolerant and anoxia tolerant vertebrates.. Environmental stress and gene regulation.

[17] Smith AT, Foggin JM (1999). The plateau pika (Ochotona curzoniae) is a keystone species for biodiversity on the Tibetan Plateau.. Animal Conservation.

[18] Du JZ, Li QF (1982). Effects of simulated hypoxia acclimation on organism, organ and hematology in Ochotona curzoniae and rats.. Acta Theriologica Sinica.

[19] Du JZ, Li QF, Chen XG (1984). Effect of simulated altitude on liver of Ochotna curzoniae and rats.. Acta Zoologica Sinca.

[20] Li QF, Sun RY, Huang CX, Wang ZW, Liu XT (2001). Cold adaptive thermogenesis in small mammals from different geographical zones of China.. Comp Biochem Physiol A Mol Integr Physiol.

[21] Wang JM, Zhang YM, Wang DH (2006). Seasonal thermogenesis and body mass regulation in plateau pikas (*Ochotona curzoniae*).. Oecologia.

[22] Ahima RS, Prabakaran D, Mantzoros C, Qu D, Lowell B (1996). Role of leptin in the neuroendocrine response to fasting.. Nature..

[23] Masuzaki H, Ogawa Y, Hosoda K, Miyawaki T, Hanaoka I (1997). Glucocorticoid regulation of leptin synthesis and secretion in humans: elevated plasma leptin levels in Cushing's syndrome.. J Clin Endoerinol Metab.

[24] Zhang Y, Proenca R, Maffei M, Barone M, Leopold L (1994). Positional cloning of the mouse obese gene and its human homologue.. Nature.

[25] Maffei M, Halaas J, Ravussin E, Pratley RE, Lee GH (1995). Leptin levels in human and rodent: measurement of plasma leptin and ob RNA in obese and weight-reduced subjects.. Nature Medicine.

[26] Piňeiro V, Casabiell X, Peino R, Garcia-Vallejo L, Dieguez C (1998). PMA inhibits both spontaneous and glucocorticoid-mediated leptin secretion by human omental adipose tissue explants *in vitro*.. Biochem Biophys Res Commun.

[27] Piňeiro V, Casabiell X, Peino R, Lage M, Camina JP (1999). Gender differences In androgen-mediated leptin secretion by human omental adipose tissue *in vitro*: Dihydrotestosterone, stanozolol, androstenedione and dehydroepiandrosterone-S inhibit leptin release in women but not in men.. J Endocrinol.

[28] Mantzoros CS, Qu D, Frederich RC, Susulic BB, Lowell E (1996). Activation of beta(3) adrenergic receptors suppresses leptin expression and mediates a leptin-independent inhibition of food intake in mice.. Diabetes.

[29] Caro JF, Sinha MK, Kolaczynski JW, Zhang PL, Considine RV (1996). Leptin: the tale of an obesity gene.. *Diabetes*.

[30] Casabiell X, Piňeiro V, Peino R, Lage M, Camina JP (1998). Gender differences in both spontaneous and stimulated leptin secretion by human omental adipose tissue *in vitro*: Dexamethasone and estradiol stimulate leptin release in women but not in men samples.. J Clin Endocr Metab.

[31] De Vos P, Saladin R, Auwerx J, Staels B (1995). Induction of ob gene expression by corticosteroids is accompanied by body weight loss and reduced food intake.. J Biol Chem.

[32] Havel PJ, Kasim KS, Mueller W, Johnson PR, Gingerich RL (1996). Relationship of plasma leptin to plasma insulin and adiposity in normal weight and overweight women: effects of dietary fat content and sustained weight loss.. J Clin Endoerinol Metab.

[33] Li XS, Wang DH (2005). Regulation of body weight and thermogenesis in seasonally acclimatized Brandt's voles (Microtus brandti).. Horm Behav.

[34] Scarpace PJ, Matheny M, Pollock BH, Tumer N (1997). Leptin increase uncoupling protein expression and energy expenditure.. Am. J Physiol Endocrinol Metab.

[35] Scarpace PJ, Michael M (1998). Leptin induction of UCP1 gene expression is dependent on sympathetic innervation.. Am. J Physiol Endocrinol Metab.

[36] Peino R, Pineiro V, Gualillo Q, Menendez C, Brenlla J (2000). Cold exposure inhibits leptin secretion in vitro by a direct non-specific action on adipose tissue.. Eur J Endocrinol.

[37] Trayhurn P, Duncan JS, Rayner DV (1996). Acute cold-induced suppression of ob (obese) gene expression in white adipose tissue of mice: mediation by the sympathetic nervous system.. Biochem J.

[38] Grusfeld A, Andre J, Mouzon SH, Berra E, Pouyssegur J (2002). Hypoxia-inducible Factor 1 Transactivates the Human Leptin Gene Promoter.. J Biol Chem.

[39] Yang J, Zhao XQ, Guo SC, Li HG, Qi DL (2006). Leptin cDNA cloning and its mRNA expression in plateau pikas (Ochotona curzonize) from different altitude on Qinghai-Tibet Plateau.. Biochem Biophys Res Commun.

[40] Posada D, Crandall KA (1998). Modeltest: testing the model of DNA substitution.. Bioinformatics.

[41] Yang ZH (1997). PAML: a program package for phylogenetic analysis by maximum likelihood.. Comput Appl Biosci.

[42] Zhang F, Basinski MB, Beals JM, Briggs SL, Churgay LM (1997). Crystal structure of the obese protein leptin-E100.. Nature.

[43] Goldman N, Yang ZH (1994). A codon-based model of nucleotide substitution for protein- coding DNA sequences.. Mol Biol Evol.

[44] Yang ZH, Kumar S, Nei M (1994). A new method of inference of ancestral nucleotide and amino acid sequences.. Genetics.

[45] Grasso P, Leinung MC, Inqher SP, Lee DW (1997). In vivo effects of leptin-related synthetic peptides on body weight and food intake in female ob/ob mice: localization of leptin activity to domains between amino acid residue 106-140.. Endocrinology.

[46] Grasso P, Leinung MC, Lee DW (1999). Epitope mapping of secreted mouse leptin utilizing peripherally administered synthetic peptides.. Regul Pept.

[47] Grasso P, White DW, Tartaqlia LA, Leinung MC, Lee DW (1999). Inhibitory effects of leptin-related synthetic peptide 116-130 on food intake and body weight gain in female C57BL/6J ob/ob mice may not be mediated by peptide activation of the long isoform of the leptin receptor.. Diabetes.

[48] Imagawa K, Numate Y, Katsuura G, Katsuurs G, Sakaguchi I (1998). Structure-function studies of human leptin.. J Bio Chem.

[49] Peelman F, Van Beneden K, Zabeau L, Iserentant H, Ulrichts P (2004). Mapping of the leptin binding sites and design of a leptin antagonist.. J Biol Chem.

[50] Zabeau L, Defeau D, Van der Heyden J, Iserentant H, Vandekerckhove J (2004). Functional analysis of leptin leptin receptor activation using a Janus kinase/signal transducer and activator of transcription complementation assay.. Mol Endocrinol.

[51] Iserentant H, Peelman F, Defeau D, Vandekerckhove J, Zabeau L (2005). Mapping of the interface between leptin and the leptin receptor CRH2 domain.. J Cell Sci.

[52] Peelman F, Iserentant H, De Smet AS, Vandekerckhove J, Zabeau L (2006). Mapping of binding site III in the leptin receptor and modeling of a hexameric leptin{middle dot}leptin receptor complex.. J Biol Chem.

[53] Futai M, Noumi T, Maeda M (1989). ATPsynthase (H+-ATPase): results by combined biochemical and molecular biological approaches.. Annu Rev Biochem.

[54] Pugh LG (1957). Resting ventilation and alveolar air on Mount Everest: with remarks on the relation of barometric pressure to altitude in mountains.. J Physiol.

[55] Peacock AJ (1998). ABC of oxygen: oxygen at high altitude.. BMJ.

[56] Dawson MR, Teichert C, Yochelson EL (1967). Lagomorph history and stratigraphic record.. Essays in Paleontology and Stratigraphy.

[57] Mead JI (1987). Quaternary records of pika, Ochotona, in North America.. Boreas.

[58] Dong G, Wang G, Chen H, Yan M, Jin J, China Society of the Qinghai-Tibet Plateau Research, editors (1995). The formation and evolution of the deserts in China and their relation to the uplifting of Qinghai-Tibet Plateau.. Qinghai-Tibet Plateau and Global Variations.

[59] Fang X, Li J, Zhu J, Zhong W, Lu W, China Society of the Qinghai-Tibetan Plateau Research, editors (1995). Environmental change of the Linxia Basin and the uplift of the Tibetan Plateau. In “Qinghai-Tibetan Plateau and Global Variations.. Qinghai-Tibetan Plateau and Global Variations.

[60] Bradford BL, Bruce MS (2000). Towards a molecular understanding of adaptive thermogenesis.. Nature.

[61] Trayhum P, Nicholls DG, Trayhum P, Nicholls DG (1986). The brown adipose tissue mitochon-drial uncoupling protein.. Brown adipose tissue.

[62] Pelleymounter MA, Cullen MJ, Baker MB, Hecht R, Winters D (1995). Effects of the obese gene product on body weight regulation in ob/ob mice.. Science.

[63] Kamohara S, Burelin R, Halaas JL, Friedman JM, Charron MJ (1997). Acute stimulation of glucose metabolism in mice by leptin treatment.. Nature.

[64] Minokoshi Y, Kim YB, Peroni OD, Fryer LG, Muller C (2002). Leptin stimulates fatty-acid oxidation by activating AMP-activated protein kinase.. Nature.

[65] Cusin J, Zakrzewska KE, Boss O, Muzzin P, Giacobino JP, Ricquier D, Jeanrenaud B, Rohner-Jeanrenaud F (1998). Chronic central leptin infusion enhances insulin-stimulates glucose metabolism and favors the expression of uncoupling proteins.. Diabetes.

[66] Muoio DM, Dohm GL, Fiedorek FT, Tapscott EB, Coleman RA (1997). Leptin directly alters lipid partitioning in skeletal muscle.. Diabetes.

[67] Rossetti L, Massillon D, Barzilai N, Vuguin P, Chen W (1997). Short term effects of leptin on hepatic gluconeogenesis and in vivo insulin action.. J Bio Chem.

[68] Cohen P, Miyazaki M, Socci ND, Hagge-Greenberg A, Liedtke W (2002). Role for stearoyl-CoA desaturase-1 in leptin-mediated weight loss.. Science.

[69] Seufert J (2004). Leptin effects on pancreatic β-cell gene expression and function.. Diabetes.

[70] Toyoshima Y, Gavrilova O, Yakar S, Jou W, Pack S (2005). Leptin improves insulin resistance and hyperglycemia in a mouse model of type 2 diabetes.. Endocrinology.

[71] Yu N, Zheng CL, Zhang YP, Li WH (2000). Molecular systematics of Pikas (*Genus Ochotona*) inferred from mitochondrial DNA sequence.. Mol Phylogenet Evol.

[72] Thompson JD, Gibson TJ, Plewniak F, Jeanmougin F, Higgins DG (1997). The Clustal X windows interface: flexible strategies for multiple sequence alignment aides by quality analysis tools.. Nucleic Acids Res.

[73] Pollastri G, Baldi P (2002). Prediction of contact maps by recurrent neural network architectures and hidden context propagation from all four cardinal corners.. Bioinformatics.

[74] Baldi P, Pollastri G (2003). The principled design of large-scale recursive neural network architectures DAG-RNNs and protein structure prediction problem.. J Mach Learn Res.

[75] Guex X, Peitsch MC (1997). SWISS-MODEL and Swiss-Pdb Viewer: An environment for comparative protein modeling.. Electrophoresis.

[76] Felsenstein J (2006). PHYLIP: Phylogeny inference package, Version 3.66..

[77] Robinson RM, Huchon D (2000). RRTree: Relative-rate tests between groups of sequences on a phylogenetic tree.. Bioinformatics.

[78] Swofford DL (2000). PAUP*: Phylogenetic analysis using parsimony (* and other methods), Version 4.0..

[79] Yang ZH, Nielsen R (2002). Codon-substitution models for detecting molecular adaptation at individual sites along specific lineages.. Mol Biol Evol.

[80] Nielsen R, Yang ZH (1998). Likelihood models for detecting positively selected amino acid sites and applications to the HIV-1 envelope gene.. Genetics.

[81] Wong WS, Yang ZH, Goldman N, Nielsen R (2004). Accuracy and power of statistical methods for detecting adaptive evolution in protein coding sequences and for identifying positively selected sites.. Genetics.

[82] Zhang J, Nielsen R, Yang ZH (2005). Evaluation of an improved branch-site likelihood method for detecting positive selection at the molecular level.. Mol Biol Evol.

[83] Yang ZH, Wong WS, Nielsen R (2005). Bayes empirical Bays inference of amino acid sites under positive selection.. Mol Biol Evol.

