# Supplementary material for: Natural Selection and Adaptive Evolution of Leptin in the Ochotona Family Driven by the Cold Environmental Stress
Source: PLoS One. 2008 Jan 23;3(1):e1472. doi: 10.1371/journal.pone.0001472 (PMC2194619; doi:10.1371/journal.pone.0001472)
Supplement: Table S1 — GenBank accession numbers of ob gene and mitochondrial cytochrome b gene of different lineages cited in this study (0.04 MB DOC) [file pone.0001472.s002.doc]

**Supporting Information 1**

| Table 7. GenBank accession numbers of *ob* gene and mitochondrial *cytochrome b* gene of different lineages cited in this study | | |
| --- | --- | --- |
| lineage | *ob* | *mytochrome b* |
| *Cyprinus carpio* | AY547279 | DQ868873 |
| *Ctenopharyngodon idella* | AY551335 | AF420424 |
| *Mus musculus* | NM_008493 | EF108336* |
| *Rattus norvegicus* | NM_013076 | AC_000022* |
| *Gorilla gorilla* | GGU72872 | NC_001645* |
| *Homo sapiens* | NM_000230 | EF657789* |
| *Ovis aries* | OAU84247 | DQ903227 |
| *Sus scrofa* | NM_213840 | EF061504 |
| *Bos taurus* | NM_173928 | DQ124416* |
| *Canis familiaris* | AB020986 | NC_002008* |
| *Ursus thibetanus japonicus* | AB255164 | AY522430 |
| *Felis catus* | AB041360 | NC_001700* |
| *Oryctolagus cuniculus* |  | AJ001588* |
| *Lepus oiostolus* |  | AY599079 |
| *Ochotona curzoniae* |  | AF273004 |
| *Ochotona nubrica* |  | AF272991 |
| *Ochotona cansus cansus* |  | AF273006 |
| *Ochotona annecten* |  | AF273008 |
| *Ochotona daurica bedfordi* |  | AF273000 |
| **denotes the sequence of complete mitochondrial genome* | | |
